# Advances in Single-Chain Nanoparticles for Catalysis Applications

**DOI:** 10.3390/nano7100341

**Published:** 2017-10-21

**Authors:** Jon Rubio-Cervilla, Edurne González, José A. Pomposo

**Affiliations:** 1 Centro de Física de Materiales (CSIC, UPV/EHU)—MPC, Materials Physics Center, Paseo Manuel de Lardizabal 5, E-20018 San Sebastian, Spain; jon.rubio015@gmail.com (J.R.-C.); edurne_gonzalez001@ehu.eus (E.G.); 2 Departamento de Física de Materiales, Universidad del País Vasco (UPV/EHU), Apartado 1072, E-20080 San Sebastian, Spain; 3 IKERBASQUE—Basque Foundation for Science, María Díaz de Haro 3, E-48013 Bilbao, Spain

**Keywords:** nanoparticles, nanocontainers, catalysts

## Abstract

Enzymes are the most efficient catalysts known for working in an aqueous environment near room temperature. The folding of individual polymer chains to functional single-chain nanoparticles (SCNPs) offers many opportunities for the development of artificial enzyme-mimic catalysts showing both high catalytic activity and specificity. In this review, we highlight recent results obtained in the use of SCNPs as bioinspired, highly-efficient nanoreactors (3–30 nm) for the synthesis of a variety of nanomaterials (inorganic nanoparticles, quantum dots, carbon nanodots), polymers, and chemical compounds, as well as nanocontainers for CO_2_ capture and release.

## 1. Introduction

Enzymes are the most efficient catalysts, known for biochemical reactions that take place in aqueous environment under crowding conditions near room temperature [1]. They have been a source of inspiration for the construction of a variety of artificial enzyme-mimic catalysts, such as macrocyclic compounds [2], star [3] and helical [4] polymers, dendrimers [5], micelles [6], vesicles [7], and polymersomes [8]. The specific, well-defined structure of enzymes accounts for its exceptional catalytic activity and specificity. Many enzymes exhibit a precision hydrophobic nano-cavity that is stabilized by hydrophobic interactions where catalysis takes place in a very efficient manner. Often, the globular structure of enzymes provides a unique local environment for the active (catalytic) site in which the biochemical reaction occurs. This folded structure is responsible for the specificity of the enzyme, and the formation and stabilization of the transition state during the transformation of reagent(s) to product(s). The folded globular structure of enzymes is often maintained via hydrogen bonding, hydrophobic interactions, and disulfide bonds. Hence, the structure of enzymes and thus its catalytic properties are highly sensitive to changes in pH, temperature, or redox potential. For efficient catalysts to occur, many enzymes also bind to the active site other small compounds (cofactors), such as metal ions or low-molecular-weight organic molecules, which work together with enzymes to enhance reaction rates [1].

**Figure 1:**
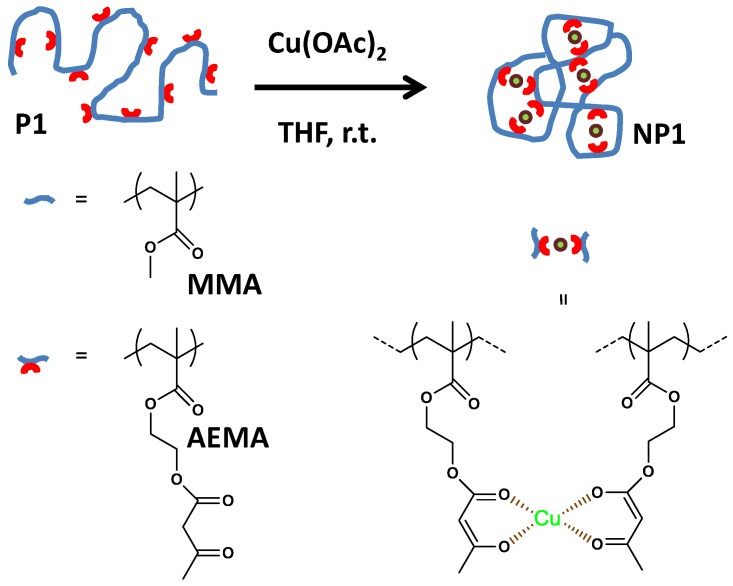
Schematic illustration of the formation of catalytic single-chain nanoparticle (SCNP) **NP1** from functional precursor polymer **P1** via intra-chain copper(II)-complexation (reprinted from [14] with permission, Copyright American Chemical Society, 2014). THF = tetrahydrofuran; MMA = methyl methacrylate; AEMA = (2-acetoacetoxy)ethyl methacrylate.

Recently, single-chain nanoparticles (SCNPs) have emerged as valuable artificial soft nano-objects, which can be efficiently endowed with useful enzyme-mimic catalytic activity (Figure 1) [9,10,11,12]. The folding of individual polymer chains to functional SCNPs is reminiscent of protein folding to its functional, native state, although current SCNPs lack the perfection in sequence, uniformity in size, and precise morphology that is found in enzymes [9]. Even so, the folding of a synthetic polymer to a collapsed state provides with one (or more) denser local packaging zone(s) where catalytic activity and selectivity can be enhanced. This should be promoted by a local polymer environment, for allowing stabilization of the transition state during catalysis. In analogy with enzymes, metal ions, or low-molecular-weight organic molecules can work together with SCNPs to enhance reaction rates. Very recently, both exceptional catalytic activity [13] and specificity [14] have been demonstrated by using metal-containing SCNPs.

**Figure 2:**
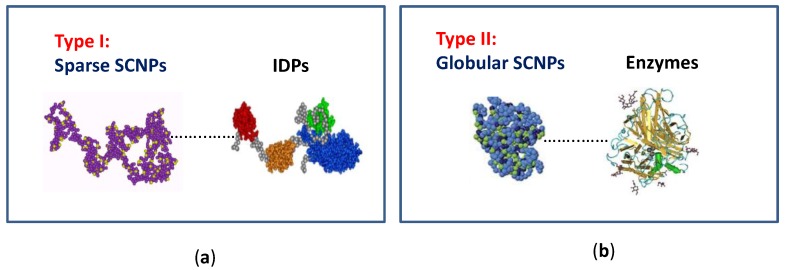
Illustration of the two limiting conformations of SCNPs: (**a**) sparse morphology resembling that typical of intrinsically disordered proteins (IDPs); and (**b**) globular morphology as often found in enzymes (reprinted from [9] with permission, Copyright Society of Chemical Industry, 2016).

Concerning the morphology of SCNPs in solution, two limiting conformations can be obtained (type I or intrinsically disordered protein-like morphology, and type II or globular enzyme-like conformation) depending on the synthesis conditions followed, and the nature of the precursor polymer employed and/or external cross-linker used (Figure 2) [9,12]. On one hand, by performing the folding/collapse process in good solvent conditions, a sparse, non-compact SCNP morphology is generally obtained as a consequence of the self-avoiding conformation of the chain under such circumstances. In this way, local compaction along the chain (giving to a pearl-necklace-like morphology) is promoted instead of compact globule formation. The sparse SCNP morphology resembles that are shown by intrinsically disordered proteins (IDPs), in which local compact zones are connected by flexible spacers (Figure 2a) [15]. Conversely, when the folding/collapse is carried out by self-assembly of neutral [16,17,18] or charged [19] amphiphilic random copolymers or by the combination of thiol-yne coupling reaction and relatively long crosslinkers [20], a globular core-shell morphology is obtained (Figure 2b). The molecular weight of the SCNP precursor polymer and its functionalization degree are essential parameters that control SCNP size.

**Figure 3:**
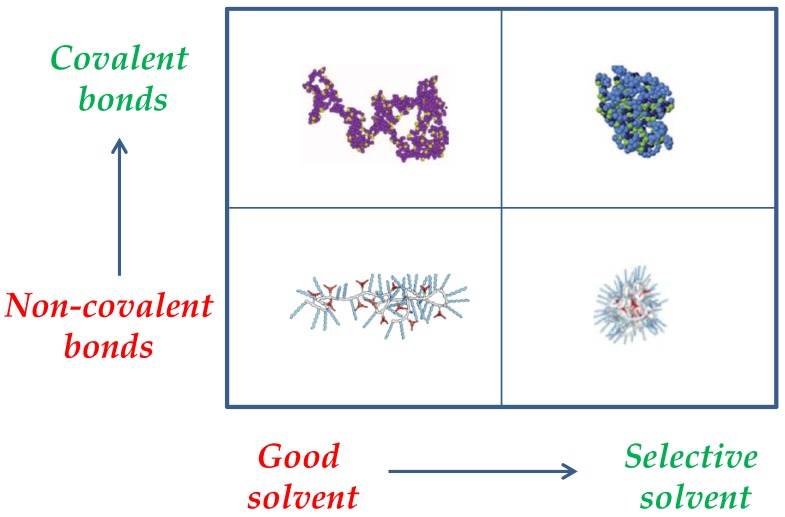
Interplay between intra-chain interactions and solvent quality on the resulting SCNP morphology and size (draws taken with permission from [9], Copyright Society of Chemical Industry, 2016 and [23], Copyright American Chemical Society, 2014).

Another way to control the degree of compaction of SCNPs is through the nature of the interactions employed to perform the folding/collapse of the precursor polymer chains, which can be permanent covalent bonds or dynamic (reversible) interactions (e.g., hydrogen bonding, metal complexation, dynamic covalent bonds). Figure 3 provides a schematic illustration of the interplay between the nature of the intra-chain interactions and solvent quality on the resulting SCNP morphology and size. A significant effort has been recently performed to predict the size reduction upon the folding/collapse of a precursor polymer chain to a SCNP via dynamic interactions [21], or covalent bonds [22] as a function of precursor molar mass (*M*) and amount of functional groups (x). In fact, covalent-bonded SCNPs in solution with similar nature, and identical values of *M* and *x* than responsive SCNPs do display, on average, a higher level of chain compaction. Moreover, in both good and selective solvents, SCNPs constructed from exactly the same precursor polymer via non-covalent interactions are expected to show a larger size than SCNPs prepared through covalent bonds [23]. The currently available intramolecular cross-linking methodologies for the synthesis of SCNPs have been the subject of a recent review [24]. Also, recent advances in the synthesis, characterization, simulations and applications of SCNPs have been compiled in a book [25].

**Figure 4:**
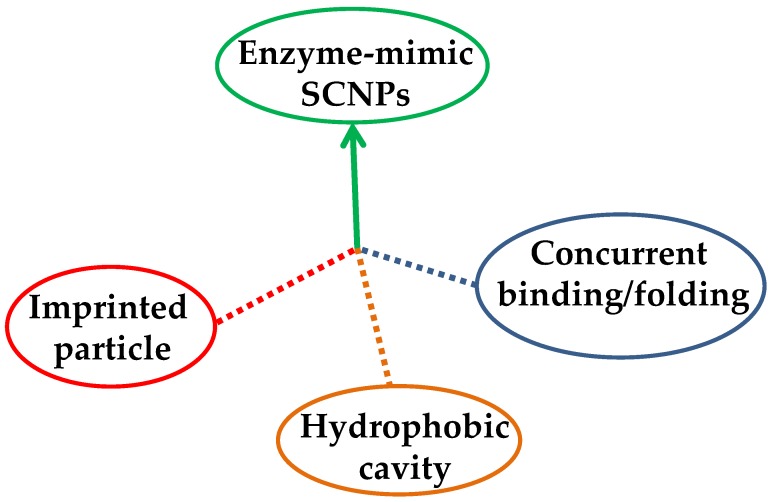
Different strategies to endow SCNPs with enzyme-mimetic activity: imprinted particle route, hydrophobic cavity approach, and concurrent binding/folding technique.

Up to now, three different approaches have been followed to endow single-molecule nanogels and SCNPs with enzyme-mimetic activity (Figure 4): (i) the “imprinted particle” route, in which the catalytic site is imprinted during the synthesis of the single-molecule nanogel via a template that is subsequently removed from each particle [26,27]; (ii) the “hydrophobic cavity” approach, in which an amphiphilic copolymer is self-folded in water and the catalytic sites are placed in the resulting hydrophobic nano-cavity [18,28,29], and (iii) the “concurrent binding/folding” strategy, which is based on the use of folding/collapsing activators playing simultaneously the dual role of cross-linkers and (nanoparticle-entrapped) catalysts [14,30,31]. While the hydrophobic cavity technique is restricted to water as a solvent, both the imprinted particle and concurrent binding/folding approaches can also be used for enzyme-like catalysis in organic solvents.

**Figure 5:**
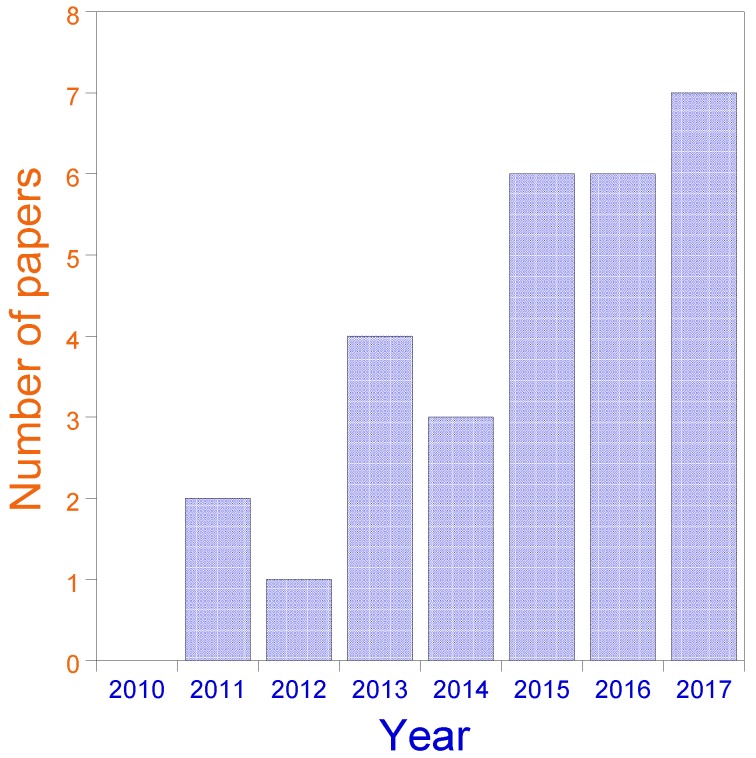
Evolution of the field of SCNPs for catalysis applications over recent years (2011–2017). Data for 2017 include only papers published until August.

The present review summarizes research in the past few years that has investigated the use of SCNPs as bioinspired, highly-efficient nanoreactors (3–30 nm) for the synthesis of a variety of nanomaterials (inorganic nanoparticles, quantum dots, carbon nanodots), polymers and chemical compounds, as well as nanocontainers for CO_2_ capture and release. The rapid evolution of this emergent field is illustrated in Figure 5.

## 2. Single-Chain Nanoparticles as Bioinspired Nanoreactors/Nanocontainers

A variety of uses of SCNPs in bioinspired catalysis have been reported by taking advantage of their folded/collapsed structure, reduced size and soft matter characteristics. Higher reactivity and selectivity has been achieved when some chemical reactions are conducted in nanoreactors. Moreover, the encapsulation of some nanomaterials generated in situ by using SCNPs as nanoreactors can provide with a better stability against aggregation or oxidation issues. In next sections, the main use of catalytic SCNPs for the synthesis of nanomaterials, polymers and chemical compounds is reviewed.

### 2.1. Synthesis of Nanomaterials

**Figure 6:**
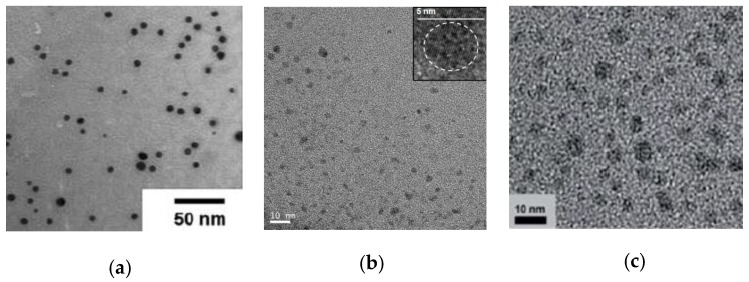
Examples of nanomaterials synthesized by using SCNPs as individual nanoreactors: (**a**) gold nanoparticles (Au-NPs); (**b**) cadmium sulfide quantum dots (CdS-QDs), dash circle in the inset shows a single CdS-QD; and, (**c**) carbon nanodots (C-NDs) (reprinted with permission from [32], Copyright Royal Society of Chemistry, 2011; [33], Copyright Wiley-VCH, 2012; and, [34], Copyright Royal Society of Chemistry, 2013).

A diversity of nanomaterials have been synthesized involving SCNPs as highly-efficient nanoreactors (Figure 6). In a pionnering work by He et al. [32], gold nanoparticles (Au-NPs) with sizes between 6 and 9 nm were synthesized in situ by using SCNPs based on *N*,*N*-dimethylaminoethyl methacrylate (DMAEMA) as nanoreactors (Figure 6a). Functional polymer precursors of high apparent molecular weight (104–110 kDa) containing 7 or 13 mol % of coumarin moieties were employed. As intramolecular cross-linking technique for the synthesis of the SCNPs, the reversible photo-induced coumarin cycloaddition was used. Dimerization of the coumarin units was carried out upon ultra-violet (UV) irradiation (*λ* = 310 nm) of a dilute solution containing the polymer precursor (1 mg/mL). The successful formation of the SCNPs was confirmed by a variety of characterization techniques, including size exclusion chromatography (SEC), viscosimetry, and differential scanning calorimetry (DSC), as well as transmission electron microscopy (TEM) measurements. Interestingly, partial photo-cleavage of the dimerized coumarin units was demonstrated upon irradiation of the SCNPs at 254 nm. The coordination ability of the tertiary amine in DMAEMA with many metals was exploited by these authors to synthesize Au-NPs in situ by using the SCNPs as nanoreactors, without any additional reductants (Figure 6a). Remarkably, the kinetics of Au-NP formation was found to strongly depend on the SCNP intramolecular cross-linking degree, which was attributed to the closer proximity of gold ions in the more compact cross-linked structures.

Qian et al. [33] reported hydrophilic SCNPs synthesized through Bergman cyclization-mediated intramolecular folding/collapse, followed by hydrogenolysis on Pd/C that were utilized as size-tunable nanoreactors to fabricate and encapsulate quantum dots (QDs) in a one-pot reaction. SCNPs were prepared from linear precursor polymers of different size (number-average molecular weight (*M*_n_): 30.3, 62.5, and 115.2 kDa) and functionalization (10 and 25 mol % of reactive enediyne groups). SEC measurements confirmed the successful formation of SCNPs, whereas the 3D morphology of the hydrophilic SCNPs on a mica surface was visualized by atomic force microscopy (AFM). As a proof of concept, photoluminescent (PL) zinc sulfide (ZnS) QDs were fabricated and encapsulated in SCNPs of different size. Smaller nanoreactors produce a single QD, each (4.1 nm in size) giving to higher PL emission intensity (quantum yield (QY) = 17% at the 310 nm excitation wavelength) while larger nanoreactors form multiple QDs each, resulting in fluorescence quenching (QY = 2%). To further investigate the generality of the method, cadmium sulfide (CdS) QDs (4.7 nm in size, Figure 6b) were synthesized and encapsulated in the smaller nanoreactors showing excellent values of quantum yield (QY = 45%).

Moreover, the use of SCNPs as sacrificial nanoreactors for the synthesis of bright photoluminescent carbon nanodots (C-NDs) was reported by the same group in 2013 (Figure 6c) [34]. C-NDs, which show excellent PL behavior, low toxicity, and environmental friendliness, are candidates to replace other PL nanomaterials for in vivo imaging and light harvesting, among other applications. Firstly, SCNPs were prepared via Bergman cyclization-mediated intramolecular folding/collapse from linear precursor polymers of different size (*M*_n_: 11.4, 35.7, and 228.4 kDa) and amount of enediyne groups (from 10 to 26.7 mol %). Secondly, the SCNPs were dispersed in a high molecular weight poly(methyl acrylate) matrix and carbonized under continuous air flow (500 °C, 30 min). Finally, the surface of the resulting C-NDs was reduced with NaBH_4_ (room temperature (r.t.), 3 h) and decorated with diamine-terminated poly(ethylene glycol) (PEG) (120 °C, 72 h). Interestingly, the optimal emission wavelength of the C-NDs was found to red-shift upon decreasing the size of the C-ND, which was attributed to the presence of an amorphous core, as supported by calculations for model compounds based on time dependent density functional theory (TD-DFT). For C-NDs with a crystalline core, just the opposite behavior was reported [35]. Smaller C-NDs showed a maximum PL emission intensity centered at 448 nm, while larger C-NDs displayed a PL peak maximum at 401 nm. The quantum yields of the C-NDs were found to be around 17% at the 340 nm excitation wavelength. The surface chemistry of the C-NDs was found to affect the QY through aggregate-induced energy/charge transfer processes, leading to a deteriorated QY.

The above examples clearly illustrate the huge possibilities of SCNPs as efficient nanoreactors for the synthesis of other complex nanomaterials.

### 2.2. Synthesis of Polymers

**Figure 7:**
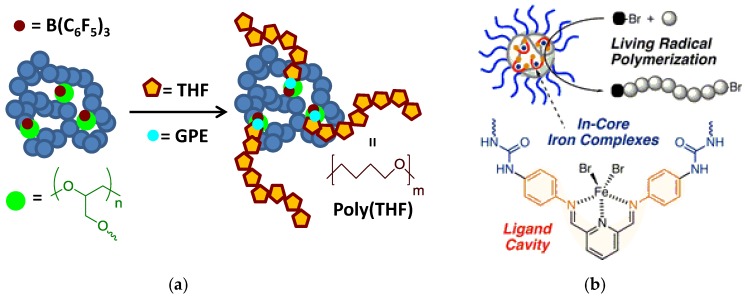
Schematic illustration of the use of SCNPs as bioinspired nanoreactors for the synthesis of polymers through: (**a**) ring-opening polymerization; and, (**b**) living radical polymerization (reprinted with permission from [30] and [29], Copyright American Chemical Society, 2013 and 2017, respectively).

SCNPs have been used as bioinspired nanoreactors for the synthesis of several polymers via ring-opening polymerization as well as controlled radical polymerization (see Figure 7).

In a pionnering work by Perez-Baena et al. [30], SCNPs were endowed with enzyme-mimetic activity by means of a new strategy called the “concurrent binding/folding” technique (see Figure 4). The technique relies on the selection of appropriate polymer precursors allowing concurrent catalyst-assisted intramolecular cross-linking and binding of the catalyst to intramolecular SCNP sites. As a proof of concept, enzyme-mimetic SCNPs were generated from glycidylic polymers via B(C_6_F_5_)_3_-assisted intramolecular ring-opening polymerization, and B(C_6_F_5_)_3_ binding to oxygen-containing functional groups (ether, carbonyl) of the SCNPs through B^…^O interactions (Figure 7a). Different SCNPs were generated with a range of hydrodynamic radii form 1.5 to 20 nm based on copolymers of benzyl methacrylate or cyclohexyl methacrylate and gycidyl methacrylate. The enzyme-mimetic activity resulted from the immobilization of the catalyst used to promote the folding/collapse in multiple, compartmentalized internal nanoparticle sites that were accessible to reagents. Tuning the global SCNP size, which presumably influences the size, composition, number, and placement of catalytic cavities, was found to have a significant effect on reaction kinetics during catalysis. Remarkably, these SCNPs showed polymerase-like activity toward tetrahydrofuran (THF) via THF ring opening polymerization activated exclusively by the presence of small amounts of glycidyl phenyl ether (GPE), a compound that played the role of cofactor. By working at low SCNP concentrations (0.3 to 2 mg/mL) and moderate reaction times (6 to 48 h), poly(THF-co-GPE) copolymers of high molecular weight (55 to 150 kDa) and very low content of GPE (<3%) but with relatively high polydispersity index (2.2 to 3.2) were synthesized.

In a further work by the same group [18], the polymerase activity displayed by metalloenzymes [36,37,38,39] was taken as inspiration to prepare copper-containing SCNPs mimicking their globular morphology and living radical polymerization activity. These bioinspired water-borne single-chain globules were constructed through intramolecular copper complexation using amphiphilic random copolymers (47.1–113.6 kDa) that are decorated with different amounts of functional groups (12–33 mol %). Interestingly, these SCNPs allowed the controlled/living radical polymerization of different water soluble monomer to be carried out under reductive conditions. A comparison of the efficiency of these bioinspired SCNPs to that of *Laccase* enzyme for the synthesis of poly(oligoethylene glycol methyl ether methacrylate) (polyOEGMA) under exactly the same reaction conditions is illustrated in Table 1 (see reference [37] for details).

In addition to polyOEGMA, high molecular weight poly(acrylic acid) (polyAA) (*M*_n_ = 115.5 kDa, *Ð* = 2.8) and poly(*N*-isopropyl acrylamide-*co*-acrylic acid) (polyNIPAM-*co*-AA) (*M*_n_ = 50.7 kDa, *Ð* = 1.8) were synthesized using these SCNPs as artificial metalloenzymes. The efficiency of the SCNPs for the synthesis of polyNIPAM-*co*-AA is compared in Table 2 with the efficiency of cysteine (Cys)-blocked bovine *Hemoglobin* for the synthesis of poly(*N*-isopropyl acrylamide) (polyNIPAM), showing the better control offered by the SCNPs over the polydispersity index of the resulting macromolecules, as well as the increased initiator efficiency.

**Figure 8:**
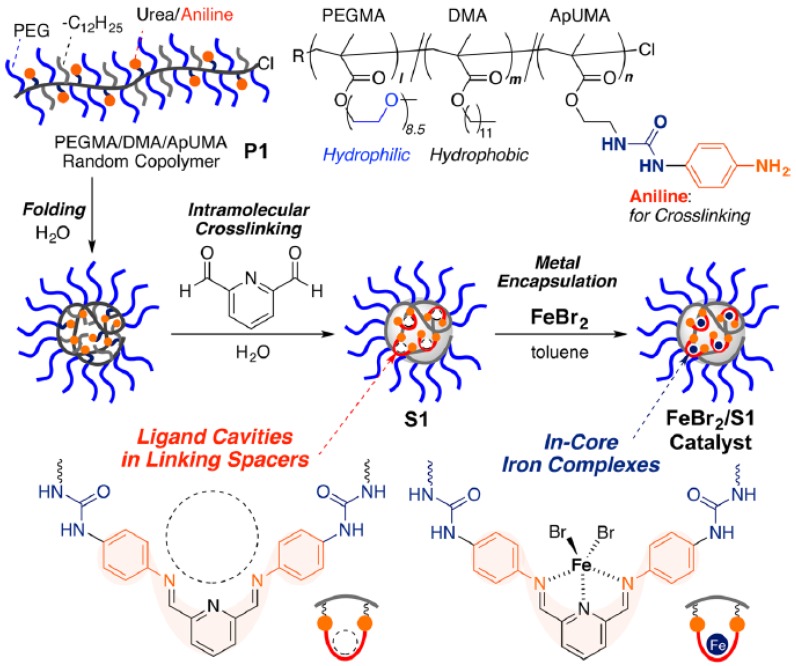
Schematic illustration of the preparation of iron-containing SCNPs of high activity for the efficient living radical polymerization of various methacrylates by using amphiphilic random terpolymers containing aniline reactive groups. Intramolecular cross-linking was promoted by imine formation, which was followed by iron complexation to the resulting bis(imino)pyridine ligand cavities (reprinted from [29] with permission, Copyright American Chemical Society, 2017).

**Figure 9:**
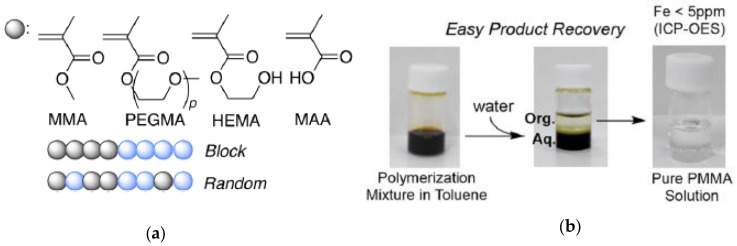
Different alkyl methacrylate monomers employed for the synthesis of block and random copolymers using iron-containing SCNPs as enzyme-mimetic catalysts (**a**); and easy recovery of nearly pure product (Fe content < 5 ppm) after polymerization (**b**) (reprinted from [29] with permission, Copyright American Chemical Society, 2017).

Recently, iron-containing SCNPs of high activity, stability, and functionality tolerance (e.g., acid, hydroxyl, and air) have been prepared by Azuma et al. [29] and employed for the efficient living radical polymerization of various alkyl methacrylate monomers (Figure 8). SCNPs were prepared from an amphiphilic terpolymer (*M*_n_ = 71.9 kDa, *Ð* = 1.29) with 22 mol % of aniline reactive groups showing a hydrodynamic radius of 6 nm in water. Firstly, 2,6-pyridinedicarboxaldehyde was used as bifunctional cross-linker of the self-folded terpolymer in water, giving rise upon intramolecular cross-linking to the generation of bis(imino)pyridine ligand cavities. Secondly, coordination of FeBr_2_ into the available SCNP bis(imino)pyridine ligand cavities was carried out via iron complexation. UV spectroscopy measurements of the SCNPs and model iron catalysts supported that the bis(imino)pyridine units effectively coordinate FeBr_2_ to form bis(imino)pyridine iron complexes. The low and reversible redox potential of the iron-containing SCNPs was ideal for their use as catalyst for living radical polymerization. As a proof of concept, the SCNPs were applied as catalyst to the living radical polymerization of methyl methacrylate (MMA) (Figure 9a) in toluene with a bromide initiator giving to well-controlled polyMMA of narrow molecular weight distribution (conversion = 90%, *M*_n_ = 4.9 kDa, *Ð* = 1.1, Br end functionality = 94%). Interestingly, the iron-containing SCNPs were effectively removed after MMA polymerization by simply washing the reaction medium with water to give bilayer solutions. The upper organic phase containing polyMMA was found to be colorless. Upon solvent evaporation, pure polyMMA was obtained (Fe content < 5 ppm) (Figure 9b).

When reused in a second catalyst cycle, the iron-containing SCNPs showed similar catalytic properties although slightly slower polymerization kinetics, which was attributed to partial oxidation of the iron catalyst during the first cycle. To illustrate the general applicability of the iron-containing SCNPs as catalyst for controlled radical polymerization, a variety of methacrylate monomers, including polyethylene glycol methyl ether methacrylate (PEGMA), 2-hydroxyethyl methacrylate (HEMA), and methacrylic acid (MAA) were successfully polymerized. Also, random and block copolymer architectures were prepared using these SCNPs as highly-efficient catalyst (Figure 9a).

Given the good results obtained with SCNPs when used as enzyme-mimetic nano-objects for the synthesis of well-defined polymers, an increasing research activity in this field is expected in the future, including the evaluation as catalyst of several metallo-containing SCNPs that were recently synthesized [40,41,42,43,44].

### 2.3. Synthesis of Chemical Compounds

Many different chemical compounds have been synthesized using SCNPs as bioinspired nanoreactors. In fact, the possibility to tune the SCNP size offers many possibilities for endowing SCNPs with enzyme-mimetic activity both in organic and aqueous media. Size can be controlled through: (i) appropriate selection of molecular weight and number of reactive groups in the precursor polymer; (ii) type of intramolecular cross-linking chemistry (covalent bonds, dynamic interactions); and, (iii) solvent selection (good solvent, selective solvent).

The first attempt to mimic the active (catalytic) site of enzymes with a single-molecule nanogel of 40 kDa was carried out by Wulff et al. [26] through the use of an “imprinted particle” technique. The catalytic site was imprinted during the synthesis of cross-linked single-molecule particles via a diphenyl phosphate template that was subsequently removed from each particle. These soft nanoparticles with a size similar to those of enzymes showed Michaelis–Menten kinetics for carbonate hydrolysis as observed in its natural counterparts although with a very low value of turnover frequency (TOF = number of moles of substrate that a mole of catalyst can convert per unit time [45]) (Table 3). The “imprinted particle” approach was subsequently adopted by Njikang et al. [27] to demonstrate the superior enantioselectivity and binding capacity against chiral amino acid derivatives of imprinted SCNPs when compared to conventional imprinted micelles.

The use of the “hydrophobic cavity” approach for the development of catalytic SCNPs in water was pioneered by Terashima et al. [28]. Catalytically active SCNPs were prepared through self-folding of an amphiphilic random terpolymer and post-loading of Ru(II) catalyst via ligand exchange with 2–3 Ru atoms per particle [46,47]. The resulting SCNPs were evaluated as catalysts for the transfer hydrogenation of ketones in the presence of sodium formate as the hydrogen source showing TOF values around 20 h^−1^ (Table 3). The same systems were found to be uniquely active in the oxidation of secondary alcohols in the presence of *t*-BuOOH as the oxidant, showing TOF values as high as 600 h^−1^ [48], which was ascribed to the significant “concentrator effect” giving to a local increase of the concentration of substrates around the catalytic centers. Interestingly, high selectivity toward more hydrophobic secondary alcohol was observed, which was attributed to the hydrophobic interior of the compartmentalized SCNP catalyst system. This hydrophobic selectivity was also observed in the reverse reaction, the transfer hydrogenation [48]. When benzene-1,3,5-tricarboxamide (BTA) functional groups capable to produce helical stacked self-assemblies via multiple hydrogen bonds were incorporated into the SCNPs, they were found to not improve the catalytic properties of the SCNPs [46,47].

On a further work by the same group, the same principle of self-folding of amphiphilic random copolymers was employed to produce water-soluble organocatalytic SCNPs containing L-proline catalytic centers (5 mol %) in the hydrophobic SCNP core [49]. These organocatalytic SCNPs were used as catalyst for the aldol reaction of cyclohexanone and *p*-nitrobenzaldehyde in water with a moderate TOF of 8 h^−1^ (Table 3), a diastereomeric excess up to 94%, and a moderate enantiomeric excess up to 70%. When BTA functionalized L-proline was incorporated to a BTA-based SCNP via molecular recognition, a higher stereoselectivity was observed [57]. Structural and surface hydration properties resembling that of enzymes were found for these water-soluble organocatalytic SCNPs [58]. However, further research is needed to understand why a retarded surface water diffusivity of the organocatalytic SCNPs is critical for activity.

Subsequently, Palmans, Meijer, and coworkers reported on a modular synthetic platform for the construction of functional SCNPs for catalysis applications [50]. BTA-based amphiphilic SCNPs were prepared using the post-polymerization functionalization of poly(pentafluorophenyl acrylate). Ligand-containing SCNPs capable of coordinating to Cu(I) and Pd(II) were prepared, which accelerated azide-alkyne cycloaddition reactions and catalyzed depropargylation reactions, respectively (Table 3). Interestingly, these reactions were found to proceed in phosphate buffer at physiological pH and at low substrate concentrations which render this kind of SCNPs promising systems to function in complex media such as cellular environments [46].

**Figure 10:**
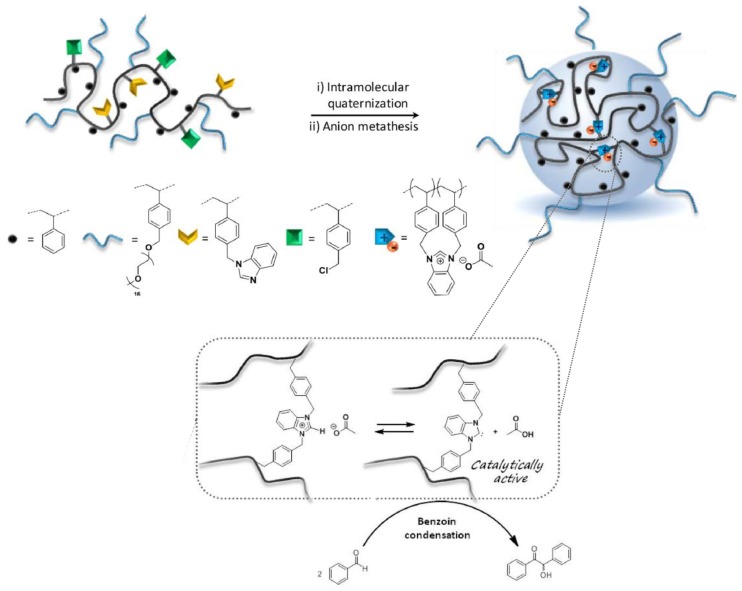
Schematic illustration of the formation of imidazolium acetate-containing SCNPs via intramolecular quaternization and anion metathesis from a statistical copolymer composed of four different comonomer units, including styrene, grafted poly(ethylene oxide) chains, and antagonist benzimidazol- and chlorobenzyl-based units, and the use of these SCNPs for the organocatalyzed benzoin condensation (adapted from [51] with permission, Copyright American Chemical Society, 2017).

Recently, the “hydrophobic cavity” approach has also been adopted by Lambert et al. [51] for the construction of imidazolium acetate-containing SCNPs, which generated SCNP-supported *N*-heterocyclic carbenes (NHCs) for organocatalyzed benzoin condensation. A statistical copolymer composed of four different comonomer units, including styrene, grafted poly(ethylene oxide) chains, and antagonist benzimidazol- and chlorobenzyl-based units was synthesized as SCNP precursor (Figure 10). The organocatalytic SCNPs (hydrodynamic diameter < 10 nm) were formed in two steps: (i) intramolecular quaternization in THF at 80 °C for 48 h; and (ii) anion metathesis in dry methanol at 40 °C for 24 h using potassium acetate. The SCNPs were tested for the organocatalyzed benzoin condensation, by generating NHCs in THF at 80 °C thanks to the ability of acetate anions to interact with protons in *C*_2_-position of benzimidazolium rings upon heating, giving rise to compartmentalized NHCs. With SCNPs with 10 mol % of supported NHCs (based on the benzimidazolium content), conversions in benzoin around 65% were achieved after 24 h of reaction time, demonstrating the in situ generation of polymer-supported NHC organocatalysts. However, the catalytic activity of these SCNPs was found to be lower than other polymer-supported NHCs, which was attributed to a non-optimized copolymer structure.

**Figure 11:**
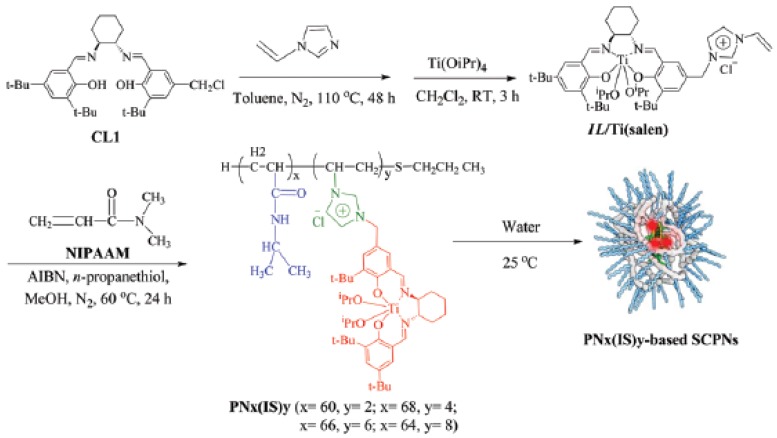
Formation via self-folding in water of poly(*N*-isopropyl acrylamide)-based SCNPs containing a chiral salen Ti^IV^ complex in the core for asymmetric sulfoxidation in water of alkyl phenyl sulfides (reprinted from [52] with permission, Copyright Royal Society of Chemistry, 2017). CL1 = (*R*,*R*)-*N*-(3,5-di-tert-butylsalicylidene)-*N*′-(3-tert-butyl-5-chloromethyl-salicylidene)-1,2-cyclohexanediamine; *IL*/Ti(salen) = vinyl imidazolium ionic liquid-modified chiral salen Ti^IV^ complex; NIPAAM = *N*-isopropylacrylamide; AIBN = azobisisobutyro-nitrile.

A further report of successful use of the “hydrophobic cavity” approach for bioinspired catalysis in water was made by Zhang et al. [52]. Firstly, thermoresponsive *N*-isopropyl acrylamide (NIPAm)-based random copolymers decorated with hydrophobic Ti(salen) pendants were prepared showing chirality by CD measurements due to the structure-forming motif of the Ti(salen) moiety employed (Figure 11). The average hydrodynamic sizes were 15.8, 11.9, 8.8, and 6.5 nm for SCNPs containing 3.2, 5.6, 8.3, and 11.5 mol % of Ti(salen) pendants. Consequently, the size of the SCNPs gradually decreased upon increasing the content of the hydrophobic Ti(salen) monomer in the precursor copolymer. Secondly, these SCNPs were evaluated as enzyme-mimetic catalysts for the enantioselective oxidation of various sulfides in water to the corresponding sulfoxides. These enzyme-mimic SCNPs were capable of significantly accelerating the asymmetric sulfoxidation in the water of methyl aryl sulfides, as well as other alkyl phenyl sulfides when compared to a traditional chiral Ti(salen) complex. Moreover, only 0.5 mol % loading of catalyst was sufficient to give excellent conversion of methyl phenyl sulfide (85–99%) in 1 h, with high chemo- (89–95%) and enantio-selectivity (95–98%). Conversely, under identical conditions, a traditional chiral Ti(salen) complex gave only 48% of conversion, 65% of chemoselectivity, and 76% of enantio excess (ee). In particular, the ee values observed for electron-rich substrates (>95% ee) represented the best results obtained so far in Ti(salen) complexes. When a batch of alkyl phenyl sulfides with various lengths of the alkyl chain was investigated, the catalytic efficiency and selectivity increased upon increasing the number of atoms of the alkyl chain. As an example, *n*-hexyl phenyl sulfide was almost quantitatively transformed to (*R*)-*n*-hexyl phenyl sulfoxide within 50 min, with 99% of chemoselectivity, and 99% of ee value. The enzyme-mimetic characteristics of these SCNPs were attributed to: (i) shielding of the catalytic sites from the surrounding aqueous environment (i.e., “site isolation”); and, (ii) confined catalysis in the compartmentalized SCNP that effectively concentrates the substrates within the hydrophobic catalytic core enhancing the reaction rate and selectivity (i.e., “confinement”). Remarkably, due to their thermo-responsive properties these SCNPs were easily recovered after reaction by simple separation upon heating at 80 °C and reused up to seven times without significant loss in activity and selectivity.

Concerning the “concurrent binding/folding” technique to endow SCNPs with enzyme-mimetic activity, it was first demonstrated in a pioneering work by Perez-Baena et al. [30]. Catalytic SCNPs were constructed from copolymer precursors containing glycidylic functional groups through B(C_6_F_5_)_3_-assisted intramolecular ring-opening polymerization and concurrent B(C_6_F_5_)_3_ binding to oxygen-containing functional groups (ether, carbonyl) of the SCNPs via B^…^O interactions (Figure 7a). The observed enzyme-mimetic catalytic activity resulted from the immobilization of catalytic B(C_6_F_5_)_3_ molecules in multiple, compartmentalized internal nanoparticle sites that were accessible to reagents. After precipitation in hexane and further drying under vacuum, the isolated SCNPs displayed reductase enzyme-mimetic activity for reactions carried out in inert organic solvents, such as dried halogenated solvents, benzene or toluene. The catalytic activity, however, was absent when reactions were performed in solvents that form adducts with B(C_6_F_5_)_3_, such as acetonitrile, dimethyl sulfoxide (DMSO), dimethyl formamide (DMF), or alcohols. The reductase activity of these SCNPs was evaluated in a model reaction involving the reduction of α-diketones to silyl-protected 1,2-diols, carried out in dichlorometane at r.t. Remarkably, the smallest SCNPs (hydrodynamic radius = 1.5–2 nm) showed the maximum catalytic activity (TOF = 5880 h^−1^) with a typical 2-fold decrease in reaction time when compared to the largest ones (Table 3).

**Figure 12:**
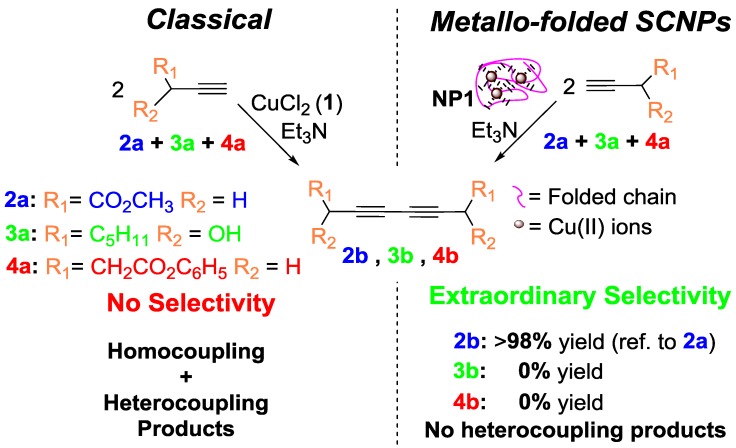
SCNPs synthesized via the “concurrent binding/folding” approach based on metallo-folded polymer chains containing complexed Cu(II) ions, showing catalytic specificity during the oxidative coupling of mixtures of chemically related terminal acetylene substrates (reprinted from [14] with permission, Copyright American Chemical Society, 2014).

In a subsequent work by the same group [14], the concurrent binding/folding strategy was utilized to synthesize catalytic SCNPs based on metallo-folded polymer chains containing complexed Cu(II) ions, showing catalytic specificity during the oxidative coupling of mixtures of chemically related terminal acetylene substrates. In this work, the intrachain cross-linking of an appropriate poly(methyl methacrylate) (PMMA)-based precursor containing acetoacetoxy ligand units towards Cu ions was used to concurrently fold the individual chains to SCNPs and endow the resulting SCNPs with catalytic activity and substrate specificity through the binding of the Cu ions to the ligand units. The resulting SCNPs approached the size of natural enzymes displaying catalytic specificity a low concentration of Cu(II) ions during the oxidative coupling of mixtures of chemically related terminal alkyne substrates (Figure 12), which cannot be achieved with classical catalysts like CuCl_2_, Cu(OAc)_2_, or Cu(acac)_2_ [14]. The specificity of the SCNPs was attributed to the presence of multiple, compartmentalized local catalytic sites surrounded by an environment of methyl methacrylate units allowing an optimum transition state stabilization for the preferred substrate. A solvent-based strategy for tuning the spatial distribution of catalytic sites in metallo-folded SCNPs has recently been reported by combining results from SEC, small-angle X-ray scattering (SAXS), and molecular dynamics (MD) simulations [59]. However, further research is needed to investigate how the internal structure of metallo-folded SCNPs affects both catalytic activity and selectivity.

Mono- and bi-metallic organometallic SCNPs containing Rh(I), Ir(I), and Ni(0) atoms based on polycycloocta-1,5-diene precursor polymers were synthesized by means of the concurrent binding/folding method by Mavila et al. [53]. Ir(I)-based SCNPs showed a catalytic efficiency during reduction of secondary amines and allylation of benzophenone similar to that of small-molecule models (Table 3). Unexpectedly, Rh(I)-based SCNPs gave biphenyl as a product during cross-coupling reactions between 4-nitrobenzaldehyde and phenyl boronic acid showing the first example of selective homo-coupling under conditions favorable to cross-coupling. Interestingly, a substantial improvement in the formation of the cross-coupled product was observed by inducing SCNP unfolding upon the addition of a NHC ligand.

Pd(II)-based SCNPs were synthesized by Willencacher et al. [54] via metal complexation using a poly(styrene) precursor polymer decorated with triarylphosphine ligand moieties. These SCNPs were used as catalysts in the Sonogashira coupling of 2-bromopyridine and phenylacetylene, showing a value of TOF = 21 h^−1^ (Table 3), slightly lower to that of a Pd(PPh_3_)_2_Cl_2_ complex used as a reference (TOF = 35 h^−1^).

Cu(I)-containing SCNPs having low toxicity and low metal loading were synthesized by Bai et al. [13] through Cu(II)-mediated intramolecular cross-linking of aspartate-containing polyofelfins in water followed by in situ reduction with sodium ascorbate. The resulting Cu(I)-containing SCNPs catalyzed azide-alkyne cycloaddition reactions with high efficiency in water showing a TOF value as high as 16,667 h^−1^ (Table 3). Remarkably, these SCNPs catalyzed intracellular click reactions inside both bacteria and mammalian cells. In one illustrative example, a fluorescent compound was synthesized inside human cancer cells via azide-alkyne cycloaddition catalyzed by the Cu(I)-containing SCNPs. In another example, an antimicrobial agent inducing strong inhibition of bacterial cell growth was synthesized inside *E. coli* via azide-alkyne cycloaddition, catalyzed by these SCNPs.

Recently, enzyme-mimetic Ni(II)-containing SCNPs have been prepared by Thanneeru et al. [55] via intramolecular Ni-thiolate coordination utilizing a PMMA-based precursor polymer functionalized with hydroxyl and thiol groups. The resulting SCNPs were highly active and selective for the photoreduction of CO_2_ to CO in DMF using TiO_2_ as a light absorber support and triethanolamine as a sacrificial electron donor, showing a TOF value as high as 2529 h^−1^ at 80 °C. Similar catalytic activity was observed by replacing DMF by CH_3_CN, which is not a good solvent for the PMMA backbone, so the binding and photoreduction of CO_2_ was not affected to a large extent by the solvation of the SCNPs. No detectable amounts of H_2_ and formate/formic acid were observed under such conditions. When compared to a metalloenzyme highly active for various conversion pathways in the biological metabolism of CO_2_, such as the carbon monoxide dehydrogenase, the Ni(II)-containing SCNPs exhibited far better stability at high temperatures and under aerobic conditions.

Recyclable homogeneous catalysts based on Pt(II)-containing SCNPs have been reported by Knöfel et al. [56]. A PS-based precursor polymer containing triarylphosphine ligand moieties along the backbone allowed for SCNP formation via the addition of Pt(1,5-cyclooctadiene)Cl_2_ in dilute solution. The resulting Pt(II)-containing SCNPs were found to be active homogeneous catalysts for the amination of allyl alcohol showing a TOF value of 16 h^−1^ with only 0.24 mol % of catalyst. These SCNPs were as active and selective as the homogeneous reference catalyst (i.e., Pt(1,5-cyclooctadiene)Cl_2_). Moreover, the Pt(II)-containing SCNPs were recovered easily after reaction by applying dialysis in methanol. After recovery, the SCNPs retained its folded structure and were found to remain catalytically active.

**Figure 13:**
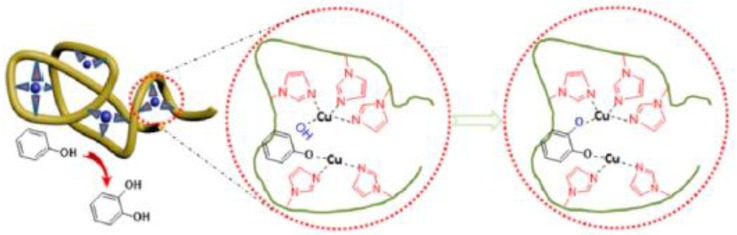
Schematic illustration of Cu-containing SCNPs employed as artificial metalloenzymes for hydroxylation reactions of phenol to catechol in water showing high selectivity (>80%) and turnover frequency (TOF) (872 h^−1^) (reprinted from [31] with permission, Copyright American Chemical Society, 2014).

Very recently, Thanneeru et al. [31] have reported the synthesis of Cu-containing SCNPs as artificial metalloenzymes for hydroxylation reactions of phenols (Figure 13). A PMMA-based prec-ursor polymer functionalized with hydroxyl and imidazole groups was prepared, and nanoparticle formation was triggered by Cu-imidazole binding at high dilution using the concurrent binding/folding technique. The SCNPs with an optimized Cu loading showed a high selectivity (>80%) and a value of TOF of 872 h^−1^ for the hydroxylation reaction of phenol to catechol (CAT) at 60 °C for 1 h in water, using H_2_O_2_ as an oxidant. Although the amount of Cu loading was found to not affect the catalytic activity (>50% conversion of phenol after 1.5 h for both SCNPs with 6.4 and 18.7 Cu atoms per particle), it affected the selectivity toward the CAT product to a large extent. Hence, a higher selectivity toward CAT (96–73%) was observed for the SCNPs with 6.4 Cu atoms per particle when compared to the SCNPs with 18.7 Cu atoms per particle (78–54%). This was attributed to the difference in flexibility of folded polymer chains that will be largely limited in the presence of large amounts of Cu-imidazole complexes. In this sense, it is worth of mention that the dynamics of the folded polymer backbone is a factor known to be critical for the activity of enzymes [60].

### 2.4. CO_2_ Capture and Release

**Figure 14:**
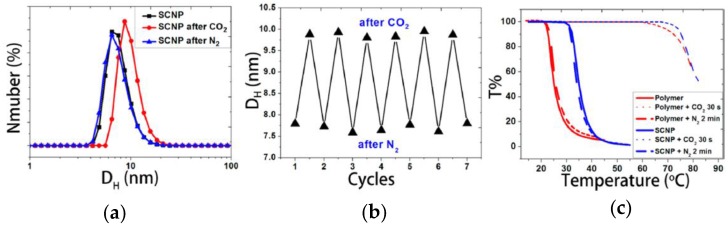
Changes in the hydrodynamic size distribution of *N*,*N*-dimethylaminoethyl methacrylate (DMAEMA)-containing SCNPs in water upon bubbling CO_2_ and N_2_ for 10 min (**a**); demonstration that the CO_2_ capture and release by these SCNPs as nanocontainers is a reversible process (**b**); and influence of CO_2_ capture and release on the thermoresponsive behavior of the precursor polymer and the corresponding SCNPs (**c**) (reprinted from [61] with permission, Copyright American Chemical Society, 2014).

Very recently, the first example of CO_2_ capture and release by SCNPs has been reported by Fan et al. [61]. Water-soluble SCNPs with a hydrodynamic size of 7.7 nm were prepared based on *N*,*N*-dimethylaminoethyl methacrylate (DMAEMA) precursor polymers containing coumarin moieties via coumarin photodimerization [32]. The average hydrodynamic size of the SCNPs in water was found to increase from 7.7 to 10 nm after passing CO_2_ for 10 min and then to decrease to the initial size after subsequently bubbling N_2_ for 10 min (Figure 14a). The CO_2_ capture and release by SCNPs was found to be a reversible process, as illustrated in Figure 14b. Bubbling CO_2_ resulted in protonation of the tertiary amine groups of the DMAEMA-containing SCNPs, making the nanoparticles more hydrophilic and promoting SCNP swelling through water absorption. Conversely, bubbling N_2_ removed CO_2_, deprotonating the tertiary amines and shrinking the SCNPs to the initial hydration level. The effect of CO_2_ capture and release on the thermoresponsive behavior of the precursor polymer and the corresponding SCNPs is depicted in Figure 14c. The precursor polymer shows a cloud point temperature of around 25 °C, which increases to 36 °C upon SCNP formation. After CO_2_ bubbling, the cloud point of both the precursor and SCNPs increased to about 75 °C as a result of protonation that enhances their water solubility. Upon N_2_ bubbling, the cloud points came back to the initial values. The use of these CO_2_ nanocontainers for synthesizing Au-NPs was also investigated by Fan et al. [61]. Remarkably, by using the DMAEMA-containing SCNPs as gas-tunable nanoreactors the rate of Au-NP formation can be sped-up or slown-down by bubbling CO_2_ or N_2_, respectively. The increased rate of Au-NP formation by bubbling CO_2_ was attributed to the swelling of the nanoreactors that facilitates access of AuCl_4_^−^ species, their association with protonated amines, and in situ reduction to zerovalent gold by nonprotonated amines.

## 3. Conclusions

Enzyme-mimetic catalysis with bioinspired SCNPs is a field of growing interest since enzymes are the most efficient catalysts known for biochemical reactions that take place in aqueous environment under crowding conditions near room temperature. The folding of individual polymer chains to functional SCNPs is reminiscent of protein folding to its functional, native state, even if current SCNPs lack the perfection in sequence, uniformity in size, and precise morphology found in enzymes. Even so, the folding of a synthetic polymer to a collapsed state provides with one (or more) denser local packaging zone(s) where catalytic activity and selectivity can be enhanced. This should be promoted by a local polymer environment allowing stabilization of the transition state during catalysis. In analogy with enzymes, metal ions or low-molecular-weight organic molecules can work together with SCNPs to enhance reaction rates.

A variety of bioinspired catalysis have been reported by taking advantage of the folded/collapsed structure, reduced size, and soft matter characteristics of SCNPs. Concerning their morphology in solution, two limiting conformations can be obtained by current synthetic methods: a sparse morphology resembling that typical of intrinsically disordered proteins (IDPs) and a globular morphology as often found in enzymes. The molecular weight of the SCNP precursor polymer and its functionalization degree are essential parameters that control SCNP size, in addition to the nature of the interactions employed to perform the folding/collapse (covalent bonds or reversible interactions) and solvent quality (good solvent, selective solvent).

Catalytic SCNPs have been prepared through three different methods: (i) the “imprinted particle” route, in which the catalytic site is imprinted via a template that is subsequently removed from each particle; (ii) the “hydrophobic cavity” approach, in which an amphiphilic copolymer is self-folded in water and the catalytic sites are placed in the resulting hydrophobic nano-cavity; and, (iii) the “concurrent binding/folding” strategy, that is based on the concurrent catalyst-assisted intramolecular cross-linking and binding of the catalyst to intramolecular SCNP sites. While the hydrophobic cavity technique is restricted to water as a solvent, both the imprinted particle and concurrent binding/folding approaches can also be used in organic solvents. In this review, recent results obtained in the use of SCNPs as bioinspired, highly-efficient nanoreactors (3–30 nm), for the synthesis of a variety of hard nanoparticles and soft materials of low and high molecular weight, as well as nanocontainers for CO_2_ capture and release have been discussed in detail.

To conclude, driven by the promising results obtained with SCNPs when used as enzyme-mimetic nano-objects for the synthesis of nanomaterials, polymers, and fine chemicals, an increasing research activity in this emergent field is expected in the near future. However, much work needs to be done to establish general rules (including type of solvents and temperature) to reach high catalytic activities, as well as to determine the added value of catalytic SCNPs against current competitors (e.g., engineered enzymes, dendrimers, metal-organic frameworks).

## Figures and Tables

**Table 1:** Comparison of *Laccase* enzyme and single-chain nanoparticles (SCNPs) as artificial metalloenzymes for the synthesis of high molecular weight polyOEGMA via controlled/living radical polymerization in water.

Catalyst Type	*c *^1^	*M*_n_ (kDa) ^2^	*Ð *^3^	*I*_eff_ (%) ^4^	Reference
*Laccase *	0.87	36.2	1.8	36	[37]
SCNPs	0.97	60.9	1.1	24	[18]

^1^ Fractional conversion. ^2^ Number-average molecular weight. ^3^ Polydispersity index. ^4^ Initiator efficiency.

**Table 2:** Comparison of cysteine (Cys)-blocked bovine *Hemoglobin* and SCNPs for the synthesis of high molecular weight poly(*N*-isopropyl acrylamide) (polyNIPAM) via controlled/living radical polymerization in water.

Catalyst Type	*c*	*M*_n_ (kDa)	*Ð*	*I*_eff_ (%)	Reference
*Hemoglobin*	0.73	200	2.2	3.2	[39]
SCNPs	0.63	50.7	1.8	6.4	[18]

**Table 3:** Summary of reactions catalyzed by unimolecular particles as bioinspired nanoreactors organized by the different strategies followed to endow them with enzyme-mimetic activity.

Reaction type ^1^	Solvent ^2^	*T* (°C) ^3^	*t* (h)	*c* (%) ^4^	TOF (h^−1^) ^5^	Reference
***Imprinted particle***						
Carbonate hydrolysis	H_2_O/MeCN	10	-	-	4.4 × 10^−3^	[26]
***Hydrophobic cavity***						
Hydrogenation of ketones	H_2_O	40	50	98	20	[28]
Oxidation of secondary alcohols	H_2_O	r.t.	7 × 10^−2^	>99	600	[48]
Aldol reaction	H_2_O	25	24	99	8	[49]
CuAAC	PBS	r.t.	0.2	>99	13	[50]
Mono-depropargylation reaction	PBS	r.t.	5	>99	0.2	[50]
Bis-depropargylation reaction	PBS	r.t.	25	>99	0.04	[50]
Benzoin condensation reaction	THF	80	24	65	0.3	[51]
Enantioselective sulfoxidation	H_2_O	25	1	99	198	[52]
***Concurrent binding/folding***						
Reduction of α-diketones	CH_2_Cl_2_	r.t.	0.1	96	5580	[30]
Alkyne dimerization	Bulk	60	8	>98	25	[14]
Reduction of secondary amines	THF	r.t.	16	>99	0.6	[53]
Allylation of benzophenone	THF	35	24	97	8	[53]
Biphenyl formation	THF	80	16	>99	0.2	[53]
Sonogashira coupling	HN(C_2_H_5_)_2_	r.t.	24	45	21	[54]
CuAAC	H_2_O	50	24	>99	16,667	[13]
Photoreduction of CO_2_	DMF	80	1.5	-	2529	[55]
Amination of allyl alcohol	C_6_D_6_	100	24	99	16	[56]
Hydroxylation of phenol	H_2_O	60	1	28	872	[31]

^1^ CuAAC = Cu(I)-catalyzed azide-alkyne cycloaddition. ^2^ MeCN = Acetonitrile; PBS = Phosphate buffer (0.01 M); THF = Tetrahydrofuran; DMF = Dimethyl formamide. ^3^ r.t. = Room temperature. ^4^ *c* = Conversion. ^5^ TOF (turnover frequency) = Amount of products (mol)/[Amount of catalyst active sites (mol)·time (h)].
